# Store-Operated Calcium Channels in the Nervous System

**DOI:** 10.1146/annurev-physiol-022724-105330

**Published:** 2025-02-03

**Authors:** Kirill S. Korshunov, Murali Prakriya

**Affiliations:** Department of Pharmacology, Northwestern Feinberg School of Medicine, Northwestern University, Chicago, Illinois, USA

**Keywords:** Ca^2+^, store-operated calcium entry, SOCE, Orai1, STIM1, neurons, astrocytes, microglia

## Abstract

Store-operated Ca^2+^ entry (SOCE) is a widespread mechanism of cellular Ca^2+^ signaling that arises from Ca^2+^ influx across the plasma membrane through the Orai family of calcium channels in response to depletion of intracellular Ca^2+^ stores. Orai channels are a crucial Ca^2+^ entry mechanism in both neurons and glia and are activated by a unique inside-out gating process involving interactions with the endoplasmic reticulum Ca^2+^ sensors, STIM1 and STIM2. Recent evidence indicates that SOCE is broadly found across all areas of the nervous system where its physiology and pathophysiology is only now beginning to be understood. Here, we review the growing literature on the mechanisms of SOCE in the nervous system and contributions to gene expression, neuronal excitability, synaptic plasticity, and behavior. We also explore the burgeoning links between SOCE and neurological disease and discuss therapeutic implications of targeting SOCE for brain disorders.

## 1. INTRODUCTION

**Figure 1:**
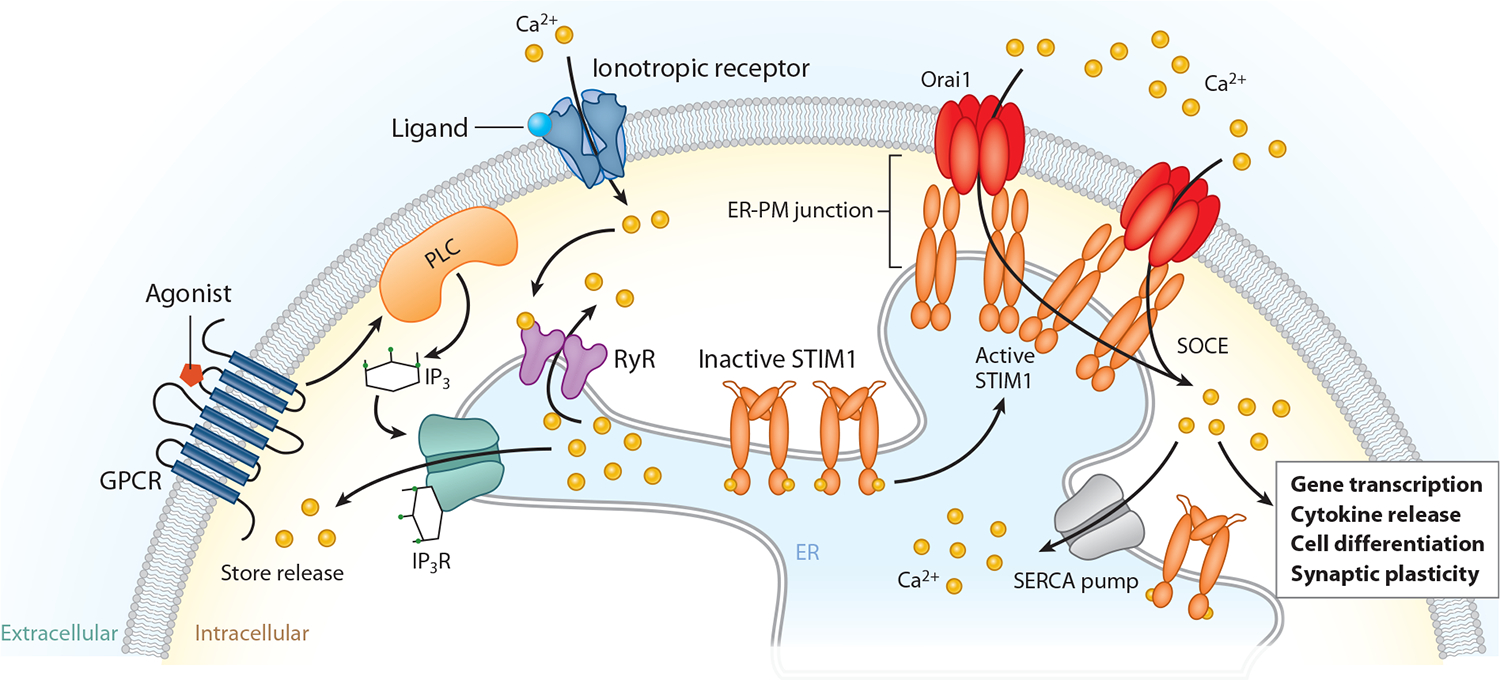
Key steps of SOCE. Stimulation of surface GPCRs or ionotropic receptors such as NMDARs leads to depletion of ER Ca^2+^ stores due to Ca^2+^ release through IP_3_Rs and/or RyRs. The ensuing fall in [Ca^2+^]_ER_ is sensed by the ER Ca^2+^ sensor, STIM1, which migrates to ER-PM junctions where it activates Orai1 channels to trigger SOCE. Abbreviations: ER, endoplasmic reticulum; GPCR, G protein–coupled receptor; IP_3_, inositol-1,4,5-triphosphate; IP_3_R, IP_3_ receptor; NMDAR, *N*-methyl d-aspartate receptor; PLC, phospholipase C; PM, plasma membrane; RyR, ryanodine receptor; SERCA, sarcoplasmic/endoplasmic reticulum Ca^2+^ ATPase; SOCE, store-operated Ca^2+^ entry; STIM1, stromal interaction molecule 1.

In most animal cells, stimulation of cell surface receptors linked to depletion of endoplasmic reticulum (ER) Ca^2+^ stores triggers Ca^2+^ influx across the plasma membrane (PM). This ubiquitous Ca^2+^ entry process, termed store-operated Ca^2+^ entry (SOCE), is tightly controlled by the Ca^2+^ concentration in the lumen of the ER and provides a direct mechanism for Ca^2+^ influx across the PM (Figure 1). The store-operated channels of immune cells, and in particular, T lymphocytes and mast cells, were the first to be characterized using electrophysiological techniques before they were found in other cells, including in the nervous system and other excitable tissues. These channels, termed Ca^2+^ release-activated Ca^2+^ (CRAC) channels, are characterized by extremely high Ca^2+^ selectivity, low unitary conductance, and low permeability to large monovalent cations (1). CRAC channels were studied for several decades before their molecular composition was identified in 2005–2006. We now know that SOCE is initiated by the stromal interaction molecule (STIM) proteins (STIM1 and/or STIM2) in the ER membrane, which sense the drop in ER Ca^2+^ that occurs following depletion of ER Ca^2+^ stores. Following store depletion, the STIM proteins directly interact with CRAC channels, which are assembled from Orai proteins, to evoke SOCE (1). STIM1 and Orai1 are the best-studied members of their respective families, and these two molecules are both necessary and sufficient to reconstitute SOCE in most cells.

The discovery of STIM1 and Orai1 spurred rapid progress in our understanding of the molecular physiology of SOCE, including the mechanisms of channel gating, ion conduction, and regulation. Genetic and biochemical approaches have also illuminated the physiological roles of store-operated Ca^2+^ channels in a variety of cell types and their contributions to human disease (2). In this review, we examine our current understanding of the physiology and pathophysiology of the SOCE mechanism in the nervous system.

## 2. HISTORY OF STORE-OPERATED Ca^2+^ ENTRY IN THE NERVOUS SYSTEM

The detailed historical accounts of the discovery of SOCE have been reviewed elsewhere (1, 3) and are not extensively covered here. Nevertheless, it is instructive to briefly highlight certain key historical milestones as the functional properties uncovered in these early studies in nonexcitable cells are also relevant to SOCE in the nervous system. The earliest indications of SOCE were found in the 1970s in rat parotid gland salivary cells. These studies uncovered a Ca^2+^ entry process across the PM that facilitated store refilling in response to adrenergic and cholinergic agonists (4). Casteels & Droogmans (5) postulated from their work in smooth muscle cells that the nature of this refilling mechanism might be a direct pathway from the extracellular space to the peripheral ER. Shortly thereafter, Putney (6, 7) synthesized these and other findings into the well-known capacitative Ca^2+^ hypothesis, which posited that the emptying of ER Ca^2+^ stores by agonists of G protein–coupled receptors (GPCRs) triggers Ca^2+^ entry into the ER to restore the depleted stores. However, the identity of the proximal stimulus responsible for Ca^2+^ entry and whether it arises from receptor-mediated inositol-1,4,5-trisphosphate (InsP_3_) or some other signal remained unclear until Putney and colleagues (8) discovered in 1989 that thapsigargin and other inhibitors of ER Ca^2+^ uptake directly activated Ca^2+^ influx without a need for receptor-mediated signaling. Soon after, electrophysiological studies by Lewis, Penner, and their colleagues (9–11) identified a store-operated Ca^2+^ current in T cells and mast cells termed the Ca^2+^ release-activated Ca^2+^ current (*I*_CRAC_) with a distinctive set of biophysical features including exquisite Ca^2+^ selectivity and low noise that set it apart from all other known Ca^2+^ channels. This Ca^2+^ entry process was subsequently renamed store-operated Ca^2+^ entry to reflect its dependence on the ER Ca^2+^ concentration and the fact that Ca^2+^ first flows into the cytosol before entering the ER.

Evidence for SOCE in the nervous system and other excitable cells emerged very shortly after the discovery of thapsigargin. In 1991, Takemura and colleagues (12) discovered a Ca^2+^ influx pathway evoked by several agents including carbachol, bradykinin, or thapsigargin in neuronal NGI08–15 and in PC12 cells with properties analogous to SOCE in rat parotid cells (8). Store depletion–activated Ca^2+^ influx was also reported in PC12 cells and SH-SY5Y neuroblastoma cells (13), and Fomina & Nowycky (14) directly recorded an inward cation current in primary chromaffin cells activated by store depletion. Soon thereafter Usachev & Thayer (15) described a caffeine-activated ryanodine-sensitive store-operated Ca^2+^ influx pathway in rat dorsal root ganglion (DRG) neurons that caused rhythmic Ca^2+^ oscillations. And in cortical and cerebellar astrocytes, store depletion by thapsigargin, cyclopiazonic acid, or agonists of metabotropic glutamate receptors evoked SOCE with pharmacological properties identical to those of CRAC channels (16, 17). Together, these early studies firmly extended the concept of SOCE to the nervous system.

**Figure 2:**
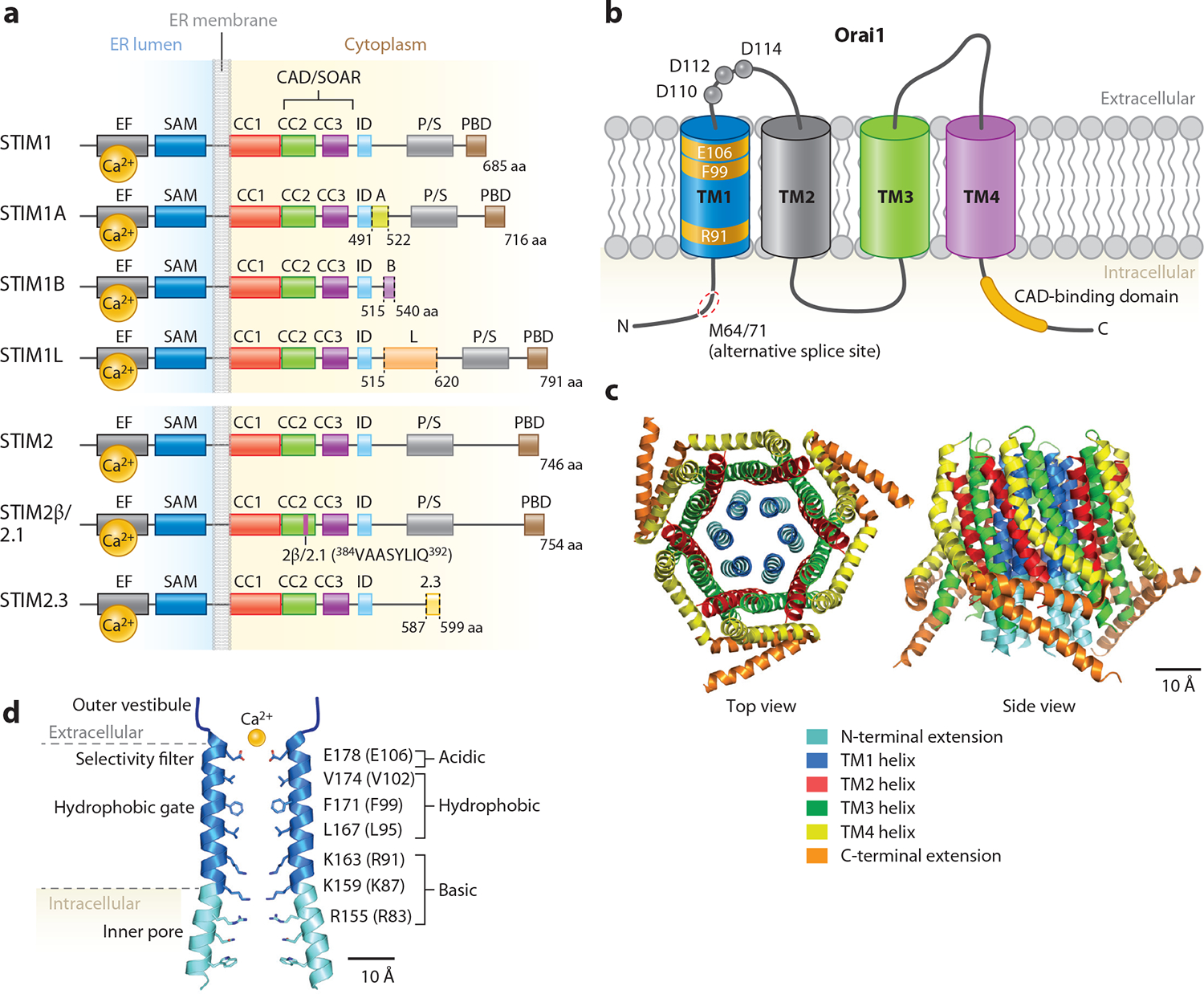
Topology and structures of STIM and Orai. (*a*) Schematics illustrating the key functional domains of STIM1 and STIM2 and their alternatively spliced isoforms. ER Ca^2+^ is sensed by the luminal EF hands. An inhibitory CC1 brake mediated by interactions between CC1 and the CAD/SOAR domain maintains STIM1 at rest when stores are full. Following store release, the intramolecular brake is released, enabling CAD/SOAR to bind to the Orai1 C terminus. The known alternative splice variants of STIM1 and STIM2 are indicated. These include STIM1A (alternative splicing in of exon 10, with a 31-aa insertion), STIM1B (alternative splicing in of exon 13B, with a 12-aa acid insertion followed by a premature stop codon after E540), and STIM1L (alternative splicing in of exon 13L, with a 106-aa insertion). STIM2 is longer than STIM1 by 61 amino acids and has EF hands with lower Ca^2+^-binding affinity. Alternative splice variants of STIM2 include STIM2β (or STIM2.1) (due to 8-aa insertion in CAD/SOAR domain by exon 9) and STIM2.3 (alternative splicing of exon 13, with a 159-aa insertion). (*b*) Topological organization of Orai1. Key residues include E106, which forms the Ca^2+^ selectivity filter; F99, which forms the hydrophobic gate; and R91, which is the locus of an immunodeficiency mutation. The C terminus contains the CAD-binding domain. (*c*) Structural organization of the Orai1 channel [Protein Data Bank identifier 4HKR (38)]. The top view shows the channel pore lined with TM1 helices and the other helices arranged in concentric circles around the pore. (*d*) Pore organization of Orai1, with the selectivity pore composed of six E106 amino acids, the hydrophobic gate lining the center of the pore, and the intracellular portion of the pore lined by basic amino acids. Abbreviations: aa, amino acid; CAD/SOAR, CRAC activation domain/STIM-Orai activating region; CC1–3, coiled-coil domains 1–3; CRAC, Ca^2+^ release-activated Ca^2+^; ID, inactivation domain; PBD, polybasic domain; P/S, proline-/serine-rich domain; SAM, sterile α motif; STIM, stromal interaction molecule; TM, transmembrane domain.

Subsequent efforts to identify the molecular basis of this channel and its activation mechanism were marked by years of frustration with many candidates presented as possible genes, only to be discarded when their biophysical properties failed to match those of I_CRAC_. This long-standing mystery was finally solved in 2005–2006 through application of forward and reverse genetic approaches, with the identification of STIM1 as the ER Ca^2+^ sensor and Orai1 as the pore-forming protein of the CRAC channel. STIM1, a 77-kDa ER membrane protein (Figure 2a), was found in RNA interference (RNAi) screens for regulators of SOCE in *Drosophila melanogaster* S2 and mammalian cells, with knockdown of STIM causing strong inhibition of SOCE and I_CRAC_ (18–20). Whereas *Drosophila* has a single STIM gene, mammals have two closely related genes, *STIM1* and *STIM2*. In resting cells, STIM1 is distributed diffusely in the bulk ER. However, ER Ca^2+^ store depletion triggers unbinding of Ca^2+^ from STIM1 EF hands that are located in the ER lumen, resulting in the redistribution of STIM1 from the bulk ER into distinct puncta localized to the membrane contact sites between the ER and PM (Figure 1). A critical line of evidence supporting the conclusion that STIM1 is the Ca^2+^ sensor for SOCE was the discovery that STIM1 EF hand mutants with impaired luminal Ca^2+^ sensing could be tricked into behaving as if stores are depleted, resulting in the formation of STIM1 puncta and causing CRAC channel activation in the absence of ER Ca^2+^ store depletion (19, 20).

The cloning of the elusive CRAC channel came soon after from studies in human patients lacking CRAC channel activity in T cells (21, 22). These patients exhibit a devastating immunodeficiency characterized by impaired T cell activation, gene expression, and cytokine synthesis, consistent with earlier evidence that CRAC channels orchestrate many aspects of lymphocyte activation and differentiation (23). Feske et al. (21) took advantage of the loss of SOCE in the patient pedigree to localize the source of the defect to a small region in chromosome 12 with ~70 genes. Simultaneously, genome-wide RNAi screens for genes involved in SOCE in *Drosophila* S2 cells independently carried out by three groups identified a novel gene as a critical mediator of *Drosophila* SOCE (21, 24, 25). A human ortholog of this gene, then annotated as *FLJ1466*, was mapped to the region on chromosome 12 identified by linkage analysis (22). Its protein, renamed Orai1, is a widely expressed 33-kDa cell surface protein with four predicted transmembrane domains (TMs), intracellular N and C termini (Figure 2b), and no significant sequence homology to other previously identified ion channels. Definitive acceptance that Orai1 was the essential pore-forming protein of the CRAC channel came from findings showing that mutation of a negatively charged glutamate in the first TM, later shown to be a part of the CRAC channel pore, markedly lowered the Ca^2+^ selectivity and divalent block of current flowing through the mutant CRAC channels (26–28). Two other closely related genes are found in humans, *C7orf19* on chromosome 7 and *MGC13024* on chromosome 16, differing from *ORAI1* primarily in the C-terminal and 3–4 loop sequences, which were renamed *ORAI2* and *ORAI3*, respectively. The human immunodeficiency linked to loss of CRAC channel activity was found to arise from a single point mutation in Orai1 (R91W) that abrogated channel activity by clogging the inner end of the pore (21, 29, 30).

## 3. GATING AND REGULATION OF ORAI CHANNELS

Activation of the CRAC channel occurs through an unusual inside-out signaling process that is unique among all ion channels. Depletion of ER Ca^2+^ stores initiates coordinated conformational changes, first in STIM1 causing it to open and extend out to interact with Orai1, followed by rearrangements in Orai1 to open the channel gate and trigger Ca^2+^ entry. These features and key steps of the gating process have been reviewed elsewhere (1); therefore, here we only briefly recap the key steps of the channel activation. The important functional domains in STIM1 that regulate its activation include a sterile alpha motif (SAM) and two EF hands at the N terminus that sense changes in the ER Ca^2+^ concentration, a catalytic domain known variously as the CRAC activation domain (CAD) (31) or the STIM1-Orai1 activating region (SOAR) (32) that binds and activates Orai channels, and a terminal polybasic domain that promotes STIM1 interactions with the PM (1, 33) (Figure 2a).

In cells with replete stores, STIM1 is inactive due to interactions between CAD/SOAR and the coiled-coil 1 (CC1) domain. Work from the Lewis, Hogan, Romanin, and other laboratories (34–36) has shown that in this resting state, the inhibitory CC1 brake causes the cytoplasmic region of STIM1 to adopt a folded compact structure. Store depletion triggers dimerization of the luminal EF-SAM domains, propagating a conformational change into the C terminus that disrupts the brake, thereby exposing and freeing up CAD/SOAR. Polybasic amino acids at the end of the STIM help aggregate STIM1 at ER-PM contact sites, and the interaction of CAD/SOAR with Orai1 traps the channels at the ER-PM junctions to evoke localized Ca^2+^ entry (1) (Figure 1). This type of channel activation process, where the stimulus (ER Ca^2+^ store depletion) triggers assembly of the channel (Orai1) with its gating particle (STIM1) at the ER-PM contact sites, is unusual among ion channels (37).

The CRAC channel itself is composed of six Orai subunits, whose TMs are arranged in concentric layers around a central aqueous pore (38) (Figure 2b,c). The TM1 helices directly flank the cation-selective pore, TM2 and TM3 surround TM1 and shield it from the surrounding lipid bilayer, and TM4 is the outermost and most lipid-exposed segment. Although some uncertainty remains about the exact positioning of the side chains, cysteine scanning (29), X-ray crystal structural analysis (38), and a host of structure-function and structural studies indicate that this model of Orai is essentially correct (1, 38).

The ion conduction pathway has four distinct structural zones, with each regulating a specific aspect of the ion permeation process. A flexible, wide extracellular turret formed by the TM1–TM2 loops of each subunit greets Ca^2+^ ions as they enter the pore (Figure 2d). Several acidic residues, 18 per channel, attract cations from the bulk solution toward the mouth of the pore through electrostatic attraction. As ions move farther into the pore, they encounter the Orai1 channel selectivity filter comprising the six glutamate residues (E106 in human Orai1). Extensive structure-function and computational evidence has shown that these glutamates are essential for the CRAC channel’s exquisite Ca^2+^ selectivity (27, 28, 39). Deeper within the ion conduction pathway is the hydrophobic zone consisting of three rings of hydrophobic amino acids: V102, F99, and L95 (Figure 2d). This region is critical for pore opening, with residues V102 and F99 playing an essential role in the gating of the channel (40, 41). Ion permeation is triggered by displacement of the F99 side chains away from the ion conduction pathway, accomplished through a combination of side chain displacement via a localized helix rotation and pore dilation, which decreases the free energy barrier for ion occupancy and permeation (41, 42). Finally, the cytoplasmic end of the pore contains a cluster of positively charged amino acids, which is highly unusual for cation channels. Although the precise role of these basic residues continues to be hotly debated, functional evidence indicates they are essential for channel activity and promote gate opening by hydrating the pore (43). These features of ion permeation and gating determined for Orai1 likely also extend to Orai2 and Orai3 channels (44).

## 4. DIVERSITY AND EXPRESSION OF STIM AND ORAI PROTEINS IN THE NERVOUS SYSTEM

Mammals express two *STIM* genes, *STIM1* and *STIM2*. Although the basic features of Ca^2+^ sensing and dynamics are believed to be similar between the two variants, STIM2, unlike STIM1, is partially active in resting cells. This is due to the lower Ca^2+^ affinity of the EF hand domains of STIM2, which allow STIM2 to respond to changes in [Ca^2+^]_ER_ around resting levels (45, 46). By contrast, STIM1 responds only to stronger levels of store depletion typically resulting from stimulation of surface receptors (45, 46).

In rodents, STIM1 is well expressed in the cerebellum, especially within the Purkinje neurons (PNs) (47–49), where an alternatively spliced variant likely predominates (50) (Table 1). However, STIM2 is expressed at higher levels than STIM1 throughout most other brain regions (49, 51), with strong STIM2 expression noted especially in the mouse hippocampus and cortex (47, 49, 52). Interestingly, in contrast to mice, human STIM1 expression appears stronger than STIM2 within many of the same regions including the hippocampus and cortex, thus indicating a potentially important species difference between mice and humans [52, 53; see also The Human Protein Atlas (https://www.proteinatlas.org)].

A notable feature of the STIM proteins is alternative splicing, which substantially increases their functional diversity (Figure 2a; Table 2). An alternatively spliced long variant of STIM1 (STIM1L) with an additional 106 amino acids spliced between exons 12 and 14 in the cytosolic region of STIM1 exists in multiple tissues including the brain (54). Niemeyer and colleagues have identified several additional splice variants including STIM1A, STIM1B, STIM2.1 (also referred to as STIM2β) (50, 55–57), and STIMγ (STIM2.3) (58). The STIM1A variant, especially enriched in hippocampal astrocytes, contains an additional 31 amino acids due to splicing of exon 11.

Overexpression of STIM1A attenuates SOCE by boosting Ca^2+^-dependent inactivation of Orai1 channels (55). Paradoxically, STIM1A also increases nuclear factor of activated T cells (NFAT) activation by increasing phosphoinositide kinase activity through an unknown mechanism (55). STIM1B is a shorter STIM1 variant lacking 170 amino acids resulting from alternative splicing of a short exon (exon 13B) between exons 12 and 14. STIM1B is well expressed in the cerebellum, cortex, and hippocampus and exerts a mild dominant negative effect on conventional STIM1-mediated SOCE (50). STIM1B expression is significantly decreased in brains of patients with Alzheimer’s disease but whether this expression change is mechanistically linked to Alzheimer’s disease is unknown (50). Another variant, STIM2β (STIM2.1) contains an 8-amino-acid insertion in the CAD, which not only prevents its ability to interact with Orai1 but also confers a dominant negative effect on STIM1 function (56, 57). Although much remains to be determined, the robust expression of STIM2 in the brain, along with the presence of numerous alternatively spliced STIM2 variants, suggests that the differential expression of these variants could exert a large influence on SOCE and Ca^2+^ dynamics in the nervous system.

Orai1 is the best characterized mammalian store-operated channel, and its deletion impairs SOCE in neurons, astrocytes, and microglia from multiple regions of the brain including the hippocampus, cortex, and spinal cord (59–64). The physiological roles of Orai2 and Orai3 are much less understood, and their deletion in mice does not impair SOCE or effector functions in immune cells (65–67). On the contrary, several studies have shown that deletion of Orai2 (and Orai3 to a smaller extent) enhances SOCE in many cells, suggesting that Orai2 may function as a negative regulator of SOCE (65–68). However, one study showed that IP_3_-mediated but not ryanodine receptor (RyR)–mediated release of Ca^2+^ stores in hippocampal CA1 pyramidal cells relies on Orai2, highlighting a role for Orai2 in certain types of agonist-evoked Ca^2+^ store responses (69). Immunohistochemistry indicates moderate to strong Orai1 protein expression in multiple regions of the brain, most notably in the cerebral cortex, hippocampus, and cerebellum (70) (Table 1). Strong expression is especially seen in the neuropil and associated dendritic processes in the hippocampus, which is a key locus for synaptic plasticity, in the molecular layer, and in PNs and granule cells of the cerebellum (63, 70). Messenger RNA (mRNA) maps indicate that the expression of Orai2 is higher in the brain than that of Orai1, with strong Orai2 mRNA expression noted in the hippocampus (49–52, 71), cerebellar PNs (48), and DRG neurons (72) (Table 1; see also The Human Protein Atlas). Given the dominant negative effects of Orai2 on SOCE (65), its strong expression in the nervous system suggests that it may suppress Orai1-mediated SOCE in the cells where Orai2 and Orai1 are both expressed. It remains unclear whether the expression of the different Orai isoforms is dynamically regulated. However, the potent dominant negative effects of Orai2 on SOCE suggest the possibility that altering the Orai1/Orai2 ratio, particularly in disease states, could lead to significant changes in SOCE magnitude and cellular Ca^2+^ signaling.

Although alternatively spliced isoforms of Orai proteins have not yet been described, a short form of Orai1 is generated by translation initiation at a second site (M64/71) (73) (Figure 2b). Both forms are expressed in most human cell lines, and with heterologous expression the relative amount of each is determined by the strength of the Kozak sequence in the expression vector (73). The shorter Orai1 exhibits weaker Ca^2+^-dependent inactivation than the full-length variant (74), raising the possibility that the strength of translation at the second start site may regulate the magnitude of SOCE in a cell-specific manner.

## 5. FUNCTIONAL ORGANIZATION OF THE STORE-OPERATED Ca^2+^ ENTRY APPARATUS IN THE NERVOUS SYSTEM

Scanning electron microscopy studies show numerous sites of membrane contact between the smooth/rough ER and the PM in the soma, dendrites, and axons (75). These sites, where active Orai-STIM complexes are expected to accumulate, are of considerable interest, as they form the organizational units for SOCE. ER-PM junctions play important roles not only in Ca^2+^ homeostasis but also in lipid transfer between the two membranes and thereby sustain a variety of effector cell functions including cell signaling, membrane trafficking, and cytoskeletal dynamics (76). How membrane contact sites are organized and maintained and what roles they play in controlling the physiology of neurons are hotly investigated topics covered in several excellent reviews (76–78). The available evidence indicates that several classes of structural and tethering proteins bridge and stabilize the 10–25-nm gap between the two membranes including the extended synaptotagmins, transmembrane protein 24 (TMEM24), Kv2.1 potassium channels, junctophilins, vesicle-associated membrane protein (VAMP)-associated proteins, and their various interacting partners (76–78).

As expected from the clustering of Orai channels in this restricted diffusional space, studies in astrocytes and immune cells show that the local SOCE-mediated Ca^2+^ signal is initiated at the ER-PM contact sites and slowly spreads away from the junctions into the nearby cytosol (79, 80). A similar functional relationship between SOCE and ER-PM junctions may be present in neurons. However, it is worth noting that Ca^2+^ tunneling, a process in which Ca^2+^ enters the ER through sarcoplasmic/endoplasmic reticulum Ca^2+^ ATPase (SERCA) at ER-PM contact sites and travels along the ER lumen to be discharged by IP_3_Rs (or RyRs) at sites far from the original SOCE microdomains, may significantly extend the size of the SOCE microdomain beyond the ER-PM contact sites (81). By effectively buffering cytoplasmic [Ca^2+^]_i_ at the Ca^2+^ entry points, Ca^2+^ tunneling can promote activation of distant targets away from ER-PM contact sites without inducing global Ca^2+^ rises (82, 83).

In both neurons and astrocytes, the ER is present throughout the soma, dendrites, and astrocyte processes as a continuous yet highly reticulate structure (84). However, some compartments that are critical for synaptic integration are essentially ER free. For example, in dendritic spines, which are the sites of excitatory synaptic input, the ER is found in only 48% of mature dendritic spines and generally is seen in the large but not small spines (85). A question that arises from this organization is the precise localization of Orai and STIM proteins: Where exactly are the Orai channels located in dendrites, spines, and the presynaptic terminals? Although no anatomical evidence is currently available, focal glutamate uncaging indicates that synaptically evoked spine Ca^2+^ signals are highly dependent on the expression of Orai1 in the postsynaptic neurons (63). RyRs, whose opening elicits Ca^2+^ signals, are detected immunohistochemically in both dendrites and dendritic spines of cultured hippocampal neurons (86). This pattern of localization is consistent with functional evidence indicating that synaptic stimulation of glutamate receptors causes rapid decrease in the [Ca^2+^]_ER_ (63). Although speculative, these features suggest that the anatomy of the SOCE mechanism in the restricted spaces of dendritic spines is optimized for fast activation and signaling. In the following sections, we explore the physiological functions of SOCE arising from this organization, highlighting where relevant the specific cell biological features of the Orai/STIM machinery optimized for particular effector functions.

## 6. PHYSIOLOGICAL FUNCTIONS OF STORE-OPERATED Ca^2+^ ENTRY IN THE BRAIN

Orai channels influence cell physiology in two main ways. First, by facilitating store refilling, they help maintain ER Ca^2+^ store homeostasis and prevent the gradual depletion of Ca^2+^ stores that can happen during cell signaling. This is crucial for the repetitive release of Ca^2+^ during action potentials and sustaining Ca^2+^ oscillations in glial cells. Second, Ca^2+^ entry through Orai channels directly contributes to both local and global Ca^2+^ increases, broadening their function beyond just maintaining Ca^2+^ store homeostasis. While these features alone point to a wide range of physiological processes in the nervous system that could be regulated by SOCE, the importance of SOCE is likely even greater than previously estimated because many previous studies did not explicitly differentiate between processes activated by store release from those stimulated by SOCE that inevitably follows, instead attributing all impacted functions to store release. Thus, it is reasonable to expect many more physiological functions that are regulated by SOCE beyond those described below. An important cautionary note here is that much of our current understanding of the role of SOCE in the nervous system stems from knockout studies of particular *Orai* and *STIM* genes in mice. Because expression of particular Orai/STIM isoforms in the mouse and human brain vary significantly, extrapolating results of mouse knockout studies to humans may not be appropriate in some cases.

### 6.1. Gene Expression

**Figure 3:**
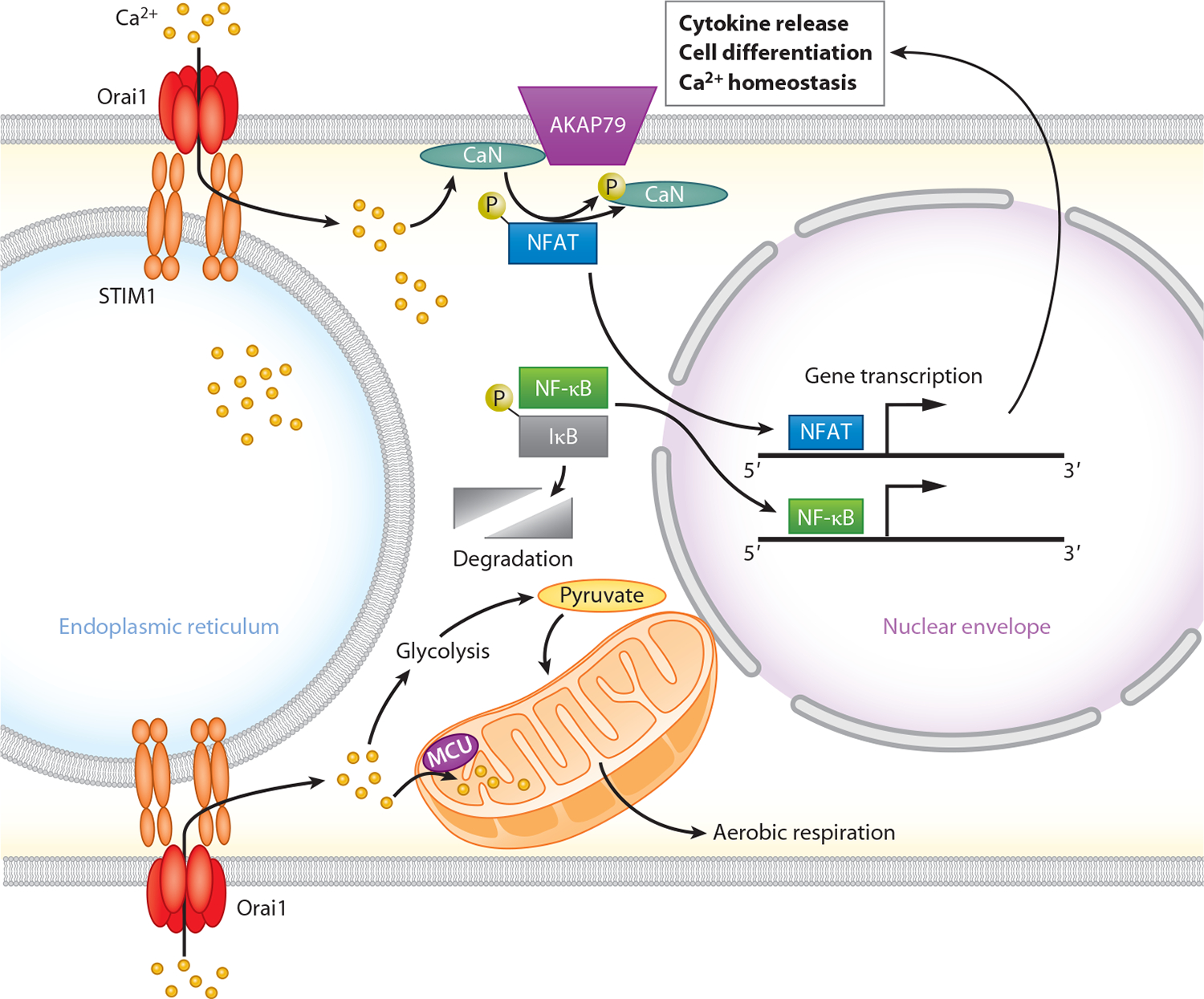
SOCE regulation of gene transcription and metabolism. Ca^2+^ influx through SOCE activates the transcription factors NFAT and NF-κB and stimulates glycolytic and mitochondrial metabolic pathways. This signaling increases transcription of cytokines and growth factors necessary for immune responses, cell proliferation, and reactivity. Abbreviations: AKAP79, A-kinase scaffolding protein 79; CaN, calcineurin; IκB, inhibitor of κB; MCU, mitochondrial Ca^2+^ uniporter; NFAT, nuclear factor of activated T cells; NF-κB, nuclear factor kappa B; SOCE, store-operated Ca^2+^ entry; STIM1, stromal interaction molecule 1.

A well-established consequence of SOCE is gene transcription stimulated by the transcription factors NFAT and nuclear factor kappa B (NF-κB) (Figure 3). NFAT activation by cellular Ca^2+^ rises is mediated by the phosphatase calcineurin, which dephosphorylates NFAT, thereby triggering its translocation into the nucleus to initiate gene transcription (87). Failure of NFAT activation in human patients with loss of function mutations in *ORAI1* or *STIM1* results in significant defects in the transcriptional synthesis of cytokines necessary for immunity and host defense resulting in immunodeficiencies, a discovery that formed the basis of the original identification of Orai1 (21). In neural progenitor cells of the subventricular zone, which produce the majority of the neuronal and glial cells of the rodent brain during embryonic development, deletion of *ORAI1* or *STIM1* causes pronounced loss of nuclear translocation of NFAT and NFAT-mediated gene expression (59). Knock-in mice carrying a nonfunctional Orai1 mutant (R93W Orai1), as well as mice with conditional deletion of *ORAI1* in the brain, exhibit reduced neural progenitor cell proliferation similar to the suppression seen with calcineurin inhibition (59). Interestingly, the differential effects of fast and slow Ca^2+^ buffers on gene expression indicate that Orai1-mediated NFAT activation is mediated by local Ca^2+^ microdomains around CRAC channels rather than bulk rises in [Ca^2+^]_i_, suggesting that calcineurin is physically coupled to CRAC channels (59). This type of functional coupling between CRAC channels and NFAT activation mediated by anchoring protein AKAP79 has been noted in other cells and enhances the specificity of Orai1-mediated gene expression (88).

RNA sequencing analysis in hippocampal astrocytes has shown that Orai1 influences a broad swath of gene expression pathways that include cellular programs related to inflammation, immunity, the cell cycle, and metabolism (89). Some of the strongest regulation occurs in interferon, adaptive immunity, and interleukin (IL) pathways controlled by the NF-κB and NFAT transcription factors (89). In line with this regulation, reporter assays support a key role for Orai1-mediated SOCE in stimulating NFAT- and NF-κB-dependent gene expression (59, 89). Importantly, deleting or knocking down Orai1 impairs regulated expression of numerous inflammatory cytokines including IL-1α, IL-6, tumor necrosis factor alpha (TNF-α), and IL-33 in hippocampal and spinal astrocytes (62, 89). Thus, SOCE-driven gene expression of inflammatory mediators is a key feature of astroglia.

New evidence indicates that, in addition to activation of Ca^2+^-dependent transcription factors, SOCE can also direct gene expression via chromatic remodeling. In *Drosophila*, where SOCE is essential for the function and development of flight neurons, Hasan and colleagues (90) showed that SOCE regulates the expression of many key ion channel and signaling genes through histone modification. Orai-mediated SOCE induced the homeobox transcription factor trithorax-like (Trl) to drive expression of the histone modifier *Set2* that encodes a histone methyltransferase (H3K36me3) in developing flight neurons (90). The ensuing histone-mediated gene expression was found to regulate a wide range of target proteins, including muscarinic acetylcholine and IP_3_Rs that are both essential for the function and development of dopaminergic neurons (90). Whether this mode of gene regulation is also present in mammalian cells remains unknown, but this finding suggests that SOCE regulates gene expression through many mechanisms beyond direct activation of Ca^2+^-dependent transcription factors.

Another interesting mode of gene regulation that likely is specific to neurons is SOCE-mediated proteasomal degradation of the transcription factor Sp4 (91). Sp4 is a neuron-specific transcription factor that influences the expression of genes important for normal dendrite patterning and synaptic plasticity. Gill and colleagues (91) found that pharmacological blockade of SOCE or knockdown of STIM1 prevented ubiquitylation and degradation of Sp4, identifying SOCE as a key regulator of Sp4-mediated gene expression via its polyubiquitylation.

Although the best-known role of STIM1 is the activation of Orai-mediated SOCE, studies have shown that STIM1 can also attenuate Ca^2+^ signaling in some cases by inhibiting voltage-gated L-type Ca^2+^ (Cav) channels (92, 93). In cultured in hippocampal neurons, Dittmer et al. (94) discovered that this function of STIM1 can suppress NFAT activation induced by strong *N*-methyl-d-aspartate (NMDA) receptor activation through glutamate uncaging. A high-frequency train of glutamate pulses delivered without external Mg^2+^ and in the presence of glycine rapidly depleted ER Ca^2+^ stores and activated STIM1, leading to the inhibition of Cav channel–mediated nuclear translocation of NFATc3 (94). Neither L-type Cav inhibition nor RNAi knockdown of STIM1 affected the structural enlargement of spines caused by high-frequency glutamate uncaging, but STIM1 knockdown reduced ER growth (94). Under physiological conditions, the role of STIM1 in gene expression may thus depend on the balance of Ca^2+^ influx from STIM1 activation of Orai-mediated SOCE and the inhibition of Cav channels.

### 6.2. Regulation of Neuronal Excitability and Spiking

Depolarizing stimuli cause neurons to fire action potentials, which form the fundamental neuronal information coding mechanism of the brain. While the basic features of action potentials are controlled by voltage-gated Na^+^ and K^+^ channels, Ca^2+^ signaling regulates excitability both directly by modulating K^+^ conductances and indirectly by regulating ion channel expression (95).

Studies in *Drosophila* have provided perhaps one of the clearest examples of the role of the SOCE in neuronal excitability in vivo (96). Double-stranded RNA knockdown of *dOrai* or *dSTIM* ablated SOCE in neuronal cultures and increased the prevalence of flightlessness in *Drosophila* (96). Further, mutants expressing a dominant-negative IP_3_R transgene in dopaminergic neurons had shorter flight bouts and decreased neuronal depolarization (97). These in vivo phenotypes were traced to reduced action potential spiking of the glutamatergic motoneurons innervating the dorsal longitudinal muscle fibers, which are critical for flight (96). Reduced excitability of flight neurons in the *dSTIM* knockdown neurons was attributed to decreased gene expression of voltage-gated Na^+^, K^+^, and Ca^2+^ channels and reduced expression of the GTPase Ral, which is necessary for flight development (98, 99). Thus, SOCE regulates neuronal excitability in *Drosophila* through multiple mechanisms converging on expression of key ion channels.

In mammals, the involvement of Orai and STIM in regulating membrane excitability seems to vary by cell type and location. In the hippocampus, one study showed that Orai1 facilitates spiking interneurons in the presence of convulsive agents such as kainic acid and pilocarpine (100). Deleting Orai1 in inhibitory neurons decreased interneuron spiking in response to kainic acid, increasing the excitatory/inhibitory ratio and enhancing seizures in response to chemoconvulsive agents (100). Likewise, Hu and colleagues (101) demonstrated that Orai1 regulates the excitability of substantia gelatinosa neurons of the spinal cord. Deleting Orai1 reduced membrane excitability of the substantia gelatinosa neurons, which was linked to a decrease in A-type K^+^ currents (101). In a related study, this group also found that deleting Orai1 reduces metabotropic glutamate receptor (mGluR)-mediated activation of the extracellular signal–regulated kinase 1/2 (ERK1/2), which in turn was associated with decreased excitability of DRG nociceptive neurons (72). The decrease in nociceptive neuron excitability was linked to relief of neuropathic pain in the Orai1 knockout mice (discussed below), suggesting that Orai1 in nociceptive neurons in the DRG and spinal cord may be an attractive target for modulation of acute and chronic pain. Another study (102) showed that in spontaneously spiking cerebellar PNs, STIM1 deletion decreased stimulus-induced spiking and increased the medium afterhyperpolarization, likely due to increased activation of SK channels arising from decreased Ca^2+^ sequestration into the ER. The reduction of spontaneous spiking observed in STIM1 knockout neurons was reproduced in wild-type neurons treated with cyclopiazonic acid, supporting the idea that STIM1 aids in refilling ER stores via SERCA and diverts free [Ca^2+^]_i_ from activating SK channels (102). Although the underlying mechanisms involved in regulation of membrane excitability seem to vary among different cell types and brain regions, these studies collectively suggest that Orai1 and STIM1 promote membrane excitability and spiking via multiple mechanisms.

### 6.3. Synaptic Transmission

**Figure 4:**
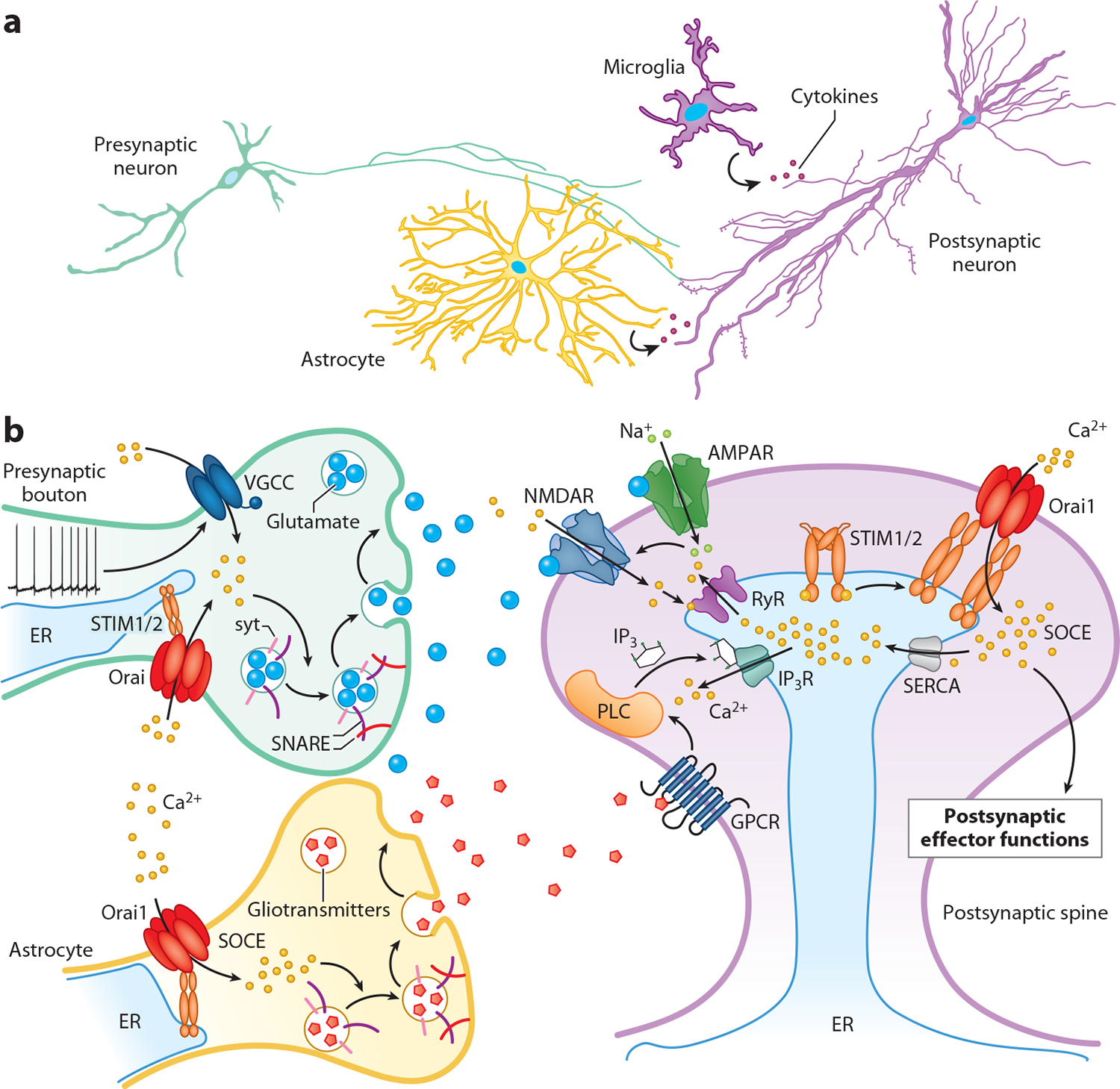
SOCE signaling at central synapses. (*a*) Schematic of the functional connections between the presynaptic nerve terminals (*green*), postsynaptic dendrites (*light purple*), astrocytes (*yellow*), and microglia (*dark purple*). (*b*) In the presynaptic bouton (*green*), SOCE activation by STIM, along with VGCC activation by action potentials, elevates presynaptic Ca^2+^ to stimulated vesicular exocytosis and neurotransmitter release. In the astrocyte processes (*yellow*) and microglia (*dark purple*), SOCE triggers gliotransmitter release and cytokine production. On the postsynaptic dendritic spine (*light purple*), glutamate activation of NMDA and metabotropic receptors initiates SOCE via CICR and IP_3_ release. Opening of RyRs and IP_3_Rs releases Ca^2+^ into the cytosol, and the ensuing Ca^2+^ store depletion activates SOCE to amplify the NMDAR-mediated Ca^2+^ signals to support long-term potentiation and other functions. Abbreviations: AMPAR, alpha-amino-3-hydroxy-5-methyl-4-isoxazolepropionic acid receptor; CICR, Ca^2+^-induced Ca^2+^ release; CRAC, Ca^2+^ release-activated Ca^2+^; ER, endoplasmic reticulum; GPCR, G protein–coupled receptor; IP_3_, inositol-1,4,5-triphosphate; IP_3_R, IP_3_ receptor; NMDAR, *N*-methyl d-aspartate receptor; PLC, phospholipase C; RyR, ryanodine receptor; SERCA, sarcoplasmic/endoplasmic reticulum Ca^2+^ ATPase; SNARE, *N*-ethylmaleimide-sensitive factor attachment protein receptor; SOCE, store-operated Ca^2+^ entry; syt, synaptotagmin; VGCC, voltage-gated Ca^2+^ channel.

In neurons, synaptic transmission is mediated by vesicular exocytosis triggered by Ca^2+^ rises in the presynaptic nerve terminal and sensed by a family of vesicle-localized Ca^2+^ sensor proteins called synaptotagmins (103). Early evidence supporting a role for SOCE vesicular exocytosis came from a study in chromaffin cells showing that depletion of intracellular Ca^2+^ stores by thapsigargin or 1,2-bis(*o*-aminophenoxy)ethane-*N,N,N,N*^′^-tetraacetic acid (BAPTA) caused slow development of a cation current that stimulated exocytosis of catecholamine containing vesicles (14). Around the same time, two studies in hippocampal brain slices showed that depletion of intracellular Ca^2+^ stores by thapsigargin or cyclopiazonic acid increased the frequency of miniature excitatory and inhibitory postsynaptic currents (mEPSCs and mIPSCs, respectively) in CA3 neurons (104, 105). These older findings were reaffirmed by subsequent studies in cultured hippocampal neurons that showed that knockdown of Orai1 or overexpression of a dominant negative Orai1 mutant reduced the frequency of mEPSCs, consistent with a role for Orai1 in regulating presynaptic vesicular fusion probability (106). More recently, a study in cultured hippocampal neurons showed that the thapsigargin-induced increase in the frequency of glutamatergic transmission is mediated by STIM2 working in conjunction with the vesicular Ca^2+^ sensor, synaptotagmin 7 (107). Interestingly, this study found that the STIM2/synaptotagmin 7 pathway enhances ER stress, suggesting that SOCE-mediated ER stress may augment neurodegeneration in brain disease (107). Collectively, these findings indicate that SOCE is a key regulator of Ca^2+^-dependent vesicular exocytosis, with Orai1 and STIM2 playing key roles in hippocampal neurons (Figure 4). As described in Section 7, Orai1 also plays a crucial role in regulating vesicular exocytosis and gliotransmitter release in astrocytes, suggesting that regulation of vesicular exocytosis is a pleiotropic SOCE function across multiple cell types.

It is worth noting that recordings from hippocampal brain slices show that *Orai1* deletion does not affect baseline excitatory or inhibitory neurotransmission in the absence of exogenous stimulation (63, 100). Conversely, overexpression of STIM1 also does not alter basal CA3–CA1 synaptic transmission and even evoked pair-pulse ratios (108). Thus, slice studies indicate that Orai1 and STIM1 do not meaningfully influence basal synaptic transmission. By contrast, many studies have found that stimulus-evoked synaptic transmission is strongly influenced by the loss of SOCE. For example, deletion of STIM1 in cerebellar PNs markedly decreased mGluR-evoked IP_3_R-dependent Ca^2+^ signals and activation of a slow depolarizing current in PNs with concomitant loss of mGluR-evoked synaptic potentials (48) despite no change in the amplitude or frequency of mEPSCs and mIPSCs in the PNs in the absence of exogenous stimulation (102). Likewise, in hippocampal CA1 neurons, deletion of Orai2 strongly diminished mGluR-evoked Ca^2+^ transients evoked by the metabotropic agonist DHPG (69). And, as described in the section below, postsynaptic Ca^2+^ responses are strongly regulated by Orai1 channels. These findings suggest that the SOCE machinery is engaged predominantly following cell stimulation by exogenous pathways that evoke store depletion leading to SOCE activation.

### 6.4. Synaptic Plasticity

Given the prominent role of Ca^2+^ signaling in synaptic plasticity and its strong links to memory and learning (109), there is great interest in understanding the physiological contributions of the Orai and STIM proteins for synaptic plasticity. The archetypal form of synaptic plasticity is long-term potentiation (LTP) at CA3–CA1 synapses in the hippocampus, a phenomenon that has been studied for decades (110) but nonetheless contains critical knowledge gaps. In this form of plasticity, intense synaptic activity evokes rhythmic bursts of Ca^2+^ signals in dendritic spines, which strengthens synapses through Ca^2+^-dependent insertion of new postsynaptic AMPA receptors in the short term and modification of dendritic spine structures over longer time scales (110, 111). A key early step in this process is Ca^2+^-mediated activation of Ca^2+^/calmodulin protein kinase (CaMKII) (111, 112), which phosphorylates numerous downstream targets necessary for both immediate and long-term changes in excitatory synaptic strength. It is generally accepted that NMDA receptors have an obligate and privileged role in triggering plasticity at this synapse (110). However, growing evidence indicates that SOCE also plays a key role in facilitating synaptic plasticity at this synapse by amplifying glutamate receptor-mediated Ca^2+^ signals.

The earliest observations linking ER Ca^2+^ store release to glutamate-evoked Ca^2+^ transients in dendrites of CA1 hippocampal neurons date back two decades ago to studies showing that NMDA receptor activation triggers Ca^2+^-induced Ca^2+^ release (CICR) from ER Ca^2+^ stores (113, 114). However, a connection to SOCE was unsuspected until later studies showed that the NMDA receptor–evoked Ca^2+^ transients in hippocampal neurons were reduced by the nonspecific SOCE antagonists 2-APB, La^3+^, and SKF96365 (104, 115). Following the identification of Orai and STIM, Segal and colleagues (106, 118) found that Orai1 knockdown or dominant-negative mutants of Orai1 impaired spine maturation and enlargement following chemical LTP stimulation in rat neuronal cultures.

More recently, Maneshi et al. (63) employed focal glutamate uncaging to activate glutamate receptors in dendritic spines and found that hippocampal neurons from conditional forebrain Orai1 knockout mice show marked loss of dendritic spine Ca^2+^ signals. A nonconducting Orai1 mutation also fails to support spine Ca^2+^ entry, indicating that Ca^2+^ permeation through Orai1 is required (63). Heterozygous Orai1 knockout mice also exhibited reduced glutamate-evoked Ca^2+^ signals (63), suggesting that this role of Orai1 may be relevant to the intellectual disability observed in human patients with heterozygous Orai1 loss due to microdeletions in chromosome 12 (116). Additionally, simultaneous measurements of ER and cytosolic Ca^2+^ signals in spines revealed a close temporal correlation between ER Ca^2+^ store depletion and Orai1-dependent cytosolic Ca^2+^ signals, with the drop in spine [Ca^2+^]_ER_ occurring approximately 100 ms before the rise in cytosolic Ca^2+^ (63). The fast kinetics of Orai1-dependent Ca^2+^ signals in the dendritic spines is strikingly different from the slower kinetics of SOCE seen in nonexcitable cells and is reminiscent of skeletal muscle, where the SOCE machinery is optimized for rapid activation and deactivation due to prepositioning of Orai1 channels near STIM1 at the triadic junctions (117). It seems likely that neuronal dendrites may have similar specializations that facilitate fast Orai1 activation at the ER-PM contact sites (Figure 4b). In line with previous studies (106, 118), the conditional Orai1 knockout mice showed deficits in several cellular indicators of synaptic plasticity induced by strong synaptic stimulation, including insertion of GluA1 receptors into the PM, spine enlargement, activation of CaMKIIα, and significant loss of LTP at the CA3–CA1 synapses (63). Collectively, these studies indicate that Orai1 and SOCE play essential roles in supporting synaptic plasticity at excitatory synapses of the hippocampus.

With respect to the STIM proteins, knockdown of STIM2 attenuates spine maturation in cultured hippocampal neurons with accompanying decreases in CAMKII activation, and these changes were proposed to underlie the aberrant plasticity seen in presenilin mutant mice that model Alzheimer’s disease (71, 119, 120). Garcia-Alvarez et al. (121) additionally found that short-hairpin RNA-mediated knockdown of STIM2, but not STIM1, impaired spine maturation in unstimulated cultured hippocampal neurons and organotypic slices, as well as forskolin-evoked phosphorylation and surface delivery of the AMPA glutamate receptor 1 subunit, GluA1. In line with these knockdown studies, mice with a conditional deletion of STIM2, but not STIM1, in excitatory neurons showed reduced phosphorylation of GluA1, cAMP response element-binding protein (CREB), and LTP in the hippocampus (122, 123). Puzzlingly, behavioral measures of working and spatial memory were unaffected in both STIM1 and STIM2 excitatory neuron-specific knockout mice (122), indicating that despite the observed alterations in spine size and GluA1 trafficking, working and spatial memory are not regulated by STIM2. However, the combined deletion of *STIM1* and *STIM2* in CaMKIIα-Cre transgenic mice paradoxically enhanced LTP at the CA3–CA1 synapse and phosphorylation of GluA1 and CREB in the hippocampus (122). The mechanisms behind the contrasting effects of deleting STIM proteins individually versus combined deletion of STIM1 and STIM2 remain unclear. However, these results raise the possibility that STIM1 and STIM2 might have opposing roles in Ca^2+^ signaling and synaptic plasticity, potentially leading to unexpected gain-of-function phenotypes in the double knockout mice.

Only a few studies have examined the contributions of Orai1 or STIM1 for other forms of synaptic plasticity including short-term synaptic plasticity and long-term depression (LTD). Pharmacological inhibition of SOCE with BTP2 inhibited mGluR-mediated LTD in cultured mouse cortical neurons, suggesting that postsynaptic SOCE plays essential roles in regulating both increases and decreases in synaptic strength (124). Conditional deletion of *Orai1* did not significantly affect paired-pulse facilitation at the CA3–CA1 synapse, a form of short-term plasticity in line with findings indicating that loss of Orai1 does not affect basal synaptic transmission in brain slices (63). However, in autaptic hippocampal neuronal cultures, overexpression of a neuronal isoform of STIM1 (STIMB) altered synaptic responses during trains of stimulation, specifically converting short-term depression that is typically seen in response to high-frequency stimulation into synaptic facilitation (50). STIM1B is predominantly localized to the presynaptic terminals, raising the interesting possibility that its expression may differentially control short-term synaptic plasticity by regulating vesicle release probability. Whether native STIM1B directly regulates presynaptic Ca^2+^ concentrations in the nerve terminals remains to be determined. Finally, although LTP was unchanged, neuron-specific overexpression of STIM1 in mice suppressed LTD induced by mGluR activation and improved contextual learning, raising the possibility that STIM1 has differing roles in LTP versus LTD (108).

### 6.5. Learning, Memory, and Cognition

As expected from the profound loss of dendritic spine Ca^2+^ signals and LTP in mice lacking Orai1 in excitatory neurons, several measures of learning and memory are depressed in these mice (63). Specifically, working and associative memory assessed by the Y-maze fear-conditioning tests are depressed in mice with neuronal-specific deletion of Orai1 (63). These behavioral changes are relatively specific to cognition, as no changes were seen in the conditional Orai1 knockout mice in balance, coordination, or general locomotion (63). Moreover, deletion of Orai1 also did not affect the ability of naïve mice to detect touch, indicating that sensorimotor behaviors are not significantly affected by loss of Orai1 in the brain (63). These results are in line with cellular measurements, indicating that the Orai1 channels are engaged predominantly in physiological contexts involving stimulus-induced synaptic plasticity rather than under basal conditions. As noted above, conditional deletion of STIM1 or STIM2 in excitatory forebrain neurons spares working memory and spatial memory (122). Yet, mice with both *STIM1* and *STIM2* deletion in CaMKIIα-expressing neurons exhibited learning deficits in the Morris water maze despite showing increases in LTP (122). Although speculative, the spatial learning defect in the *STIM1* and *STIM2* double knockout mice was hypothesized to arise from increased LTP destabilizing learning behaviors (122).

The cerebellum, crucial for balance, equilibrium, and motor coordination, is another area of the brain where ER Ca^2+^ stores and SOCE play important roles in synaptic plasticity. *Stim1* mRNA expression is higher than that of *Stim2* in PNs (48), which contrasts with the higher expression of *Stim2* in the hippocampus (125) and cortex (126). Conditional knockout of STIM1 in PNs did not alter either LTD or LTP in PNs (102). However, the PN-specific STIM1 knockout mice fail to show increases in PN excitability that normally occur in response to stimulating the parallel fibers, suggesting that intrinsic plasticity, but not long-term synaptic plasticity, is regulated by STIM1 in the cerebellum (102). Mice with PN-specific loss of STIM1 also showed deficits in the consolidation (but not acquisition) of vestibulo-ocular reflex learning (102), a process that is dependent on synaptic plasticity (127). Because the PN-specific STIM1 knockout mice show deficits in intrinsic plasticity (but not LTD or LTP), these studies suggest that STIM1 regulates consolidation of the cerebellar-dependent learning tasks that are dependent on intrinsic plasticity (102). The involvement of STIM2 and Orai channels in these and other forms of cerebellar learning (including the conditioned blink response) remain to be addressed.

## 7. SPECIALIZED FUNCTIONS OF STORE-OPERATED Ca^2+^ ENTRY IN GLIA

The glial cells of the brain, including astrocytes and microglia, do not fire action potentials and are nonexcitable. Instead, glial excitability is driven by elevations in intracellular Ca^2+^ by neurotransmitters such as glutamate and ATP. As already described in Section 6, Orai1-mediated SOCE plays an essential role in regulated gene expression in astrocytes through activation of the calcineurin-NFAT and NF-κB transcriptional pathways, which is vital for synthesis and release of inflammatory mediators underlying neuroinflammation (62, 89) (Figure 3). Likewise, Orai1- and STIM1-mediated SOCE is a prominent Ca^2+^ influx pathway in microglia necessary for agonist-mediated gene expression of inflammatory mediators and phagocytosis (62, 128–130). Interestingly, CREB, a well-known Ca^2+^-activated transcription factor in neurons with strong links to synaptic plasticity, appears to be less important in astrocytes and is insensitive to Orai1-mediated Ca^2+^ signaling (131). This underscores the point that glia likely utilize their own unique toolkit for Ca^2+^-regulated transcription, though they may share many of these pathways with neurons.

A unique astrocyte function with strong links to Ca^2+^ signaling where Orai1 plays an indispensable role is gliotransmitter release (60). In response to GPCR-mediated Ca^2+^ mobilization, astrocytes release gliotransmitters such as glycine, d-serine, GABA, and ATP to modulate the function of neighboring neurons (132). This function of astrocytes is especially critical at the tripartite synapse where astrocyte end feet are in very close proximity to pre- and postsynaptic neuronal compartments (Figure 4). A study using synapto-pHluorin to monitor single vesicle exocytosis found that activation of Orai1 stimulates vesicular exocytosis in response to a wide range of extracellular stimuli including store depletion with thapsigargin and stimulation of purinergic and protease activated receptors (60). Deletion of Orai1 in astrocytes impairs gliotransmitter release and blocked the ability of astrocytes to stimulate inhibitory neurons in the CA1 hippocampus underscoring the ability of astrocyte SOCE to regulate neural circuits (60). Astrocyte gliotransmission appears less spatially and/or temporally targeted than transmitter release from neurons, suggesting that gliotransmitter release may regulate global network activity over the course of seconds and minutes rather than milliseconds (60, 133).

Another key effector glial cell function with strong links to Ca^2+^ signaling is energy metabolism. Ca^2+^ signaling regulates energy production at multiple levels including by stimulating glycolysis, mitochondrial Ca^2+^ uptake, and aerobic respiration (134, 135). In astrocytes, genetic deletion of Orai1 impairs ATP production and the expression of critical glycolytic enzymes including hexokinase 2, which catalyzes the rate-limiting step of glycolysis (89). Loss of Orai1 signaling diminishes multiple metabolites including pyruvate, lactate, and α-ketoglutarate and alters the NADH/NAD^+^ redox balance (89). Further, octopamine, a neuronally derived trace amine, stimulates lactate production from astrocytes through Orai1-mediated SOCE and calcineurin (136). As lactate produced by astrocytes is an important metabolic substrate for energy production in neurons, loss of Orai1-mediated lactate production in astrocytes may depress neuronal functions at multiple levels.

## 8. DISEASE MODELS

The diverse roles of Orai and STIM in neurons and glia have implications for a wide range of neurological disorders. To date, however, the majority of human Orai1 and STIM1 mutations found in genetic registries have reported only links to non-neurological indications of immunodeficiency, tubular myopathy, and platelet disorders. A major limitation in determining whether mutations in Orai/STIM cause neurological dysfunctions is that germline loss-of-function mutations in Orai1 and STIM1 are often fatal at a very early age (<1 year). This limits the diagnosis of brain disorders, which are typically confirmed at later stages of development (137). Yet a growing number of entries for Orai1 and STIM1 mutations in genetic registries reveal the occurrence of brain disorders including intellectual disability, neuromuscular disorders, autism, hemorrhagic brain disorders, and others (see, e.g., https://www.ncbi.nlm.nih.gov/gtr/). A cautionary note here is that these entries do not necessarily indicate that the identified variants are directly responsible for the patient’s symptoms. As a result, preclinical animal models with conditional deletion or manipulation of Orai and STIM genes have provided much insight into the role of aberrant CRAC channel function for neurological diseases.

### 8.1. Alzheimer’s Disease

Alzheimer’s disease is highly prevalent form of dementia that disrupts normal biological aging with progressive memory loss, learning impairment, and a loss of identity. The commonly accepted causes of Alzheimer’s disease include buildup of amyloid β plaque and accumulation of hyperphosphorylated tau tangles, which are both linked to dysregulation of Ca^2+^ homeostasis in neurons and glia (138). These changes include exaggerated Ca^2+^ release from ER Ca^2+^ stores through RyRs and IP_3_Rs, elevated ER Ca^2+^ store content, attenuated SOCE, and a host of changes in neuronal excitability and neurotransmission that together incite excitotoxicity (139). Early studies of familial Alzheimer’s disease (FAD) cases noted that fibroblasts isolated from FAD mutations in the γ-secretase enzymes, presenilins (PS1 and PS2), suppressed SOCE and exaggerated Ca^2+^ release through IP_3_R and RyRs (140). One study noted that the expression of STIM1 is lower in postmortem brains of sporadic Alzheimer’s patients compared to age-matched controls (141). The decrease in STIM1 expression and SOCE was subsequently traced to γ-secretase activity of the FAD PS1 mutant, which enhanced cleavage of STIM1 and decreased the maturation of dendritic spines, a hallmark phenotype of Alzheimer’s disease (142). These alterations in spine morphology and Ca^2+^ homeostasis were rescued by overexpressing STIM1 or inhibiting the γ-secretase activity (142). STIM2 has also been shown to play a role in the process, with earlier studies indicating that mouse models of Alzheimer’s disease with either FAD-linked PS1 or amyloid precursor protein mutations suppress SOCE and STIM2 expression in hippocampal neurons, leading to changes in CaMKII activity and the density of mature spines (119, 120). Overexpression of STIM2 reversed the suppression of SOCE, phosphorylated CaMKII, and the number of mushroom spines, indicating that STIM2 also plays a key neuroprotective role in Alzheimer’s disease (119, 120). Although much remains to be understood, these complex changes in STIM expression and SOCE driven by presenilin activity may play a role in the pathogenesis of Alzheimer’s disease.

### 8.2. Epilepsy

Epilepsy is a widespread and heterogeneous brain disorder that causes unprovoked seizures due to imbalance in electrical activity in excitatory circuits in the brain (143). Human patients with heterozygous microdeletions of chromosome 12 containing the *ORAI1* gene show epilepsy and forms of intellectual disability (52, 109). In line with these case reports, conditional loss of Orai1 or STIM1 in inhibitory neurons markedly enhances seizures triggered by the chemoconvulsants, kainic acid, and pilocarpine (100). Electrophysiological interrogation of hippocampal inhibitory interneurons has shown that the chemoconvulsant-mediated increases in activity of inhibitory interneurons seen in wild-type mice was lost in the conditional Orai1 knockout mice (100). Thus, these findings suggested that SOCE plays a key role in protecting the brain against asynchronous brain activity and seizures by promoting excitability of inhibitory neurons (100). On the other hand, an in vitro study examining neuronal activity in cultures and slices found that inhibitors of broad-spectrum inhibitors of SOCE [2-APB, ML-9 1-(5-chloronaphthalenesulfonyl) homopiperazine hydrochloride] suppressed epileptic burst activity (53). Furthermore, overexpression of the Orai1 protein in the central nervous system under the control of the Thy-1 promoter caused transgenic mice to show spontaneous seizures in aged female mice (144). These complex findings underscore the need for further preclinical analysis to better understand the implications of blocking SOCE for epileptic activity and seizure induction.

### 8.3. Neurodegenerative Motor Diseases

Neurodegenerative motor diseases are a diverse group of progressive neurological diseases that destroy the function or motor neurons over time. Two such conditions where SOCE is implicated are spinocerebellar ataxias (SCAs) and Parkinson’s disease. SCAs are a heterogeneous group of motor degeneration diseases that primarily affect the cerebellum and the brain stem (145, 146). Studies in a mouse model of one subtype, termed SCA2, found that cerebellar PNs show higher basal [Ca^2+^]_i_ and increased Ca^2+^ signals in response to activation of mGluR1 receptors (147). These alterations in Ca^2+^ signaling were traced to increased IP_3_R-mediated ER store release (148). STIM1 and STIM2 expression are both altered in SCA2 models, with STIM1 showing significant increases (149). Small interfering RNA targeting of STIM1 improved motor performance of SCA2 mice and increased numbers of mushroom body spines, suggesting that unregulated influx of Ca^2+^, possibly driven through IP_3_R-mediated store release and SOCE, may contribute to the disease phenotypes of SCA in PNs (149).

Parkinson’s disease arises from the α-synuclein-induced toxicity of dopamine neurons within the substantia nigra pars compacta. A large number of studies have shown that hyperactive Ca^2+^ signaling may be a key contributing factor in the death of substantia nigra pars compacta dopamine neurons due to toxic levels of reactive oxygen species (ROS) and mitochondrial dysfunctions arising from the aberrant Ca^2+^ activity (150). Studies in induced pluripotent stem cell (iPSC)-derived neurons from Parkinson’s disease patients have found that SOCE is attenuated in dopaminergic iPSCs, which was linked to the Ca^2+^ homeostasis defects in patients with Parkinsonian mutations in PLA2g6, a phospholipase A2 enzyme involved in phosphatidyl 4,5-biphosphate metabolism. PLA2g6 was found to biochemically interact with STIM1 and loss-of-function mutations in PLA2g6-impaired SOCE (151). Although the mechanistic links between Parkinson’s disease, PLA2g6, and SOCE clearly need further clarification, these early results suggest that SOCE has a protective role in Parkinson’s disease.

### 8.4. Affective Mood Disorders

Involvement of Ca^2+^ dysregulation in affective mood disorders such as depression, bipolar disease, and schizophrenia has been considered for several decades. In particular, there is growing evidence that the pathophysiology of many affective mood disorders including depression is linked to increased brain inflammation mediated by glial cells (152). Increased microglial and astrocyte activation enhances inflammatory cytokines in the brain including that of IL-6, interferon-γ (IFN-γ), and TNF-α to produce cognitive and mood disturbances (153). Because Orai1-mediated SOCE is a positive driver of astrocyte and microglial reactivity and stimulates the generation of inflammatory mediators (89), growing evidence indicates that glial SOCE is linked to affective mood disorders. For example, a recent study found that in a model of acute depression evoked by peripheral lipopolysaccharide administration, astrocyte Orai1 knockout mice show decreases in inflammatory brain markers and protection against development of depression-like behaviors (89). Analysis of changes in excitatory and inhibitory neurotransmission in the hippocampus suggests that the pattern of changes in neurotransmission evoked by ablating Orai1 function in astrocytes resembles the effects of rapidly acting antidepressants such as ketamine (89, 154), suggesting a similar mechanism for amelioration of depression-like behaviors and implicating Orai1 as a potential therapeutic target for depression.

### 8.5. Neuropathic Pain

Several preclinical studies indicate that SOCE plays a major role in the induction and maintenance of neuropathic pain (61, 62, 72, 155). Orai1 regulation appears to involve both glia and neurons albeit through differing mechanisms (156). Hu and colleagues (62, 72) have documented multilevel alterations in the spinal cord and DRG neurons caused by nerve injury, including hyperexcitability of DRG and spinal dorsal horn neurons mediated by SOCE that appears to be dependent on suppression of A-type K^+^ channels leading to neuronal hyperexcitability and neuropathic pain. The alterations in neuronal activity in wild-type mice likely arise from neuroinflammation mediated by microglia and astrocytes (157). Inflammatory cytokines produced by microglia and astrocytes drive central sensitization in excitatory synaptic transmission in the spinal cord to cause hyperalgesia and allodynia (158). SOCE mediated by Orai1 plays a critical role in this process by regulating the transformation of microglia and astrocytes into inflammatory cell states (61). Interestingly, the effects of knocking out microglial Orai1 channels or pharmacologically blocking Orai1 are highly sex specific, with deletion of Orai1 mitigating pain hypersensitivity in male but not in female mice (61). Much remains to be understood regarding the mechanisms involved in SOCE regulation of neuropathic pain including the basis of the sex dependence and the relative contributions in neurons versus glia. However, this growing evidence portends a number of ways in which targeting Orai/STIM pathways could aid the quest for developing new therapies for inflammatory diseases affecting brain function.

## 9. CONCLUDING PERSPECTIVES

Studies in the last three decades have firmly established SOCE as a major mechanism of Ca^2+^ signaling in the nervous systems, and we now know that this pathway regulates a range of essential cellular functions in the brain including gene expression, synaptic transmission, and plasticity. Further, growing evidence indicates that SOCE contributes to brain inflammation and pathology in many models of neurological disease. Despite progress in these topics, several fundamental questions related to the mechanisms of SOCE in the nervous system remain unanswered. A key unresolved issue surrounds the functional organization of Orai and STIM proteins in neurons, astrocytes, and microglia. In which subcellular compartments are the SOCE proteins expressed? How are they organized in the presynaptic and postsynaptic compartments and in the distal processes of astrocytes, microglia, and oligodendrocytes? What are the molecular and spatial relationships of the Orai and STIM proteins with the vesicular release machinery? And what are the key regulators of SOCE in the nervous system and their underlying mechanisms? There is increasing recognition that these physiological properties need to be studied in systems that recapitulate the complex interactions and organization of different brain cells. Advances in imaging techniques (e.g., multiphoton and light sheet microscopy), three-dimensional organoid cultures, and patient-derived, iPSC-derived neurons and astrocytes will be invaluable in studying these organizational and physiological functions in a manner that is meaningful to the intact brain. Finally, recent drug discovery efforts have yielded promising new investigational drugs against Orai1 for peripheral immune disorders (159), but these are yet to be evaluated in the central nervous system. Although much remains to be done, recent research progress suggests that the coming decade could see significant advances in addressing these and related questions.

## Figures and Tables

**Table 1:** Expression of Orai and STIM isoforms in the nervous system

SOCE component	Expression	Notes	References
Orai1	Cortex, thalamus, hypothalamus, hippocampus, cerebellum, spinal cord, DRGs, choroid plexus	Low to moderate expression throughout the human brain including the hippocampus, spinal cord, and midbrain	The Human Protein Atlas;^a^ 49, 50, 63, 69, 70, 72
Orai2	Cortex, cerebellum, hippocampus, thalamus, hypothalamus, spinal cord, DRGs, choroid plexus	Orai2 mRNA expression is higher in the brain than Orai1	The Human Protein Atlas; 49–51, 69, 71, 72
Orai3	Cortex, cerebellum, hippocampus, thalamus, hypothalamus, DRGs, choroid plexus	Orai3 regulates SOCE and excitability in DRGs; other functions in the nervous system are unknown	The Human Protein Atlas; 49, 50, 72
STIM1	Cortex, hippocampus, cerebellum, thalamus, hypothalamus, DRGs, spinal cord, cortical astrocytes	Strong STIM1 expression seen throughout the human brain including hippocampus, cortex, and cerebellum	The Human Protein Atlas; 47–50, 53, 72
STIM2	Hippocampus, cortex, cerebellum, thalamus, hypothalamus, DRGs, spinal cord, choroid plexus	STIM2 expression is higher than STIM1 in the mouse but not human hippocampus and cortex	The Human Protein Atlas; 47–50, 53, 71, 72
STIM1L	Skeletal muscle, brainstem, cerebellum	Function in nervous system is unknown	54
STIM1B	Hippocampal and cerebellar neurons	Preferential presynaptic localization	50
STIM1A	Astrocytes, Sertoli cells	None	55
STIM2β/2.1	Glioblastoma, naïve CD4 and CD8 cells	None	56, 57
STIMγ/2.3	Cerebellum	None	58

a Human Protein Atlas: https://www.proteinatlas.org.

Abbreviations: DRG, dorsal root ganglion; PN, Purkinje neuron; SOCE, store-operated Ca^2+^ entry; STIM, stromal interaction molecule.

**Table 2:** STIM alternative splice variants

Splice variant	Structure	Function	References
STIM1L	Splicing in of exon 13L, causing a 106-aa insertion	Increases amount and speed of SOCE	54
STIM1B	Splicing in of exon 13B, causing a 12-aa insertion followed by a premature stop codon, resulting in overall shortening by 170 aa	Decreases SOCE, increases short-term enhancement, present in lower levels in human brains with Alzheimer’s disease	50
STIM1A	Splicing in of exon 10, causing a 31-aa insertion	Reduces SOCE, increases FCDI and NFAT translocation	55
STIM2β/2.1	Exon 9 splice, 8-aa insertion	Reduces SOCE	56, 57
STIMγ/2.3	Alternative exon 13, 159-aa deletion	Enhances SOCE	58

Abbreviations: aa, amino acid; FCDI, fast Ca^2+^-dependent inactivation; NFAT, nuclear factor of activated T cells; SOCE, store-operated Ca^2+^ entry; STIM, stromal interaction molecule.
